# Animal infection models using non‐mammals

**DOI:** 10.1111/1348-0421.12834

**Published:** 2020-08-22

**Authors:** Chikara Kaito, Kanade Murakami, Lina Imai, Kazuyuki Furuta

**Affiliations:** ^1^ Graduate School of Medicine, Dentistry, and Pharmaceutical Sciences Okayama University Okayama Japan

**Keywords:** infection model, non‐mammals, pathogenic bacteria

## Abstract

The use of non‐human animal models for infection experiments is important for investigating the infectious processes of human pathogenic bacteria at the molecular level. Mammals, such as mice and rabbits, are also utilized as animal infection models, but large numbers of animals are needed for these experiments, which is costly, and fraught with ethical issues. Various non‐mammalian animal infection models have been used to investigate the molecular mechanisms of various human pathogenic bacteria, including *Staphylococcus aureus*, *Streptococcus pyogenes*, and *Pseudomonas aeruginosa*. This review discusses the desirable characteristics of non‐mammalian infection models and describes recent non‐mammalian infection models that utilize *Caenorhabditis elegans*, silkworm, fruit fly, zebrafish, two‐spotted cricket, hornworm, and waxworm.

## 1 INTRODUCTION

Using mice as an animal infection model of anthrax, Koch first identified *Bacillus anthracis* as a human pathogen. Because *B. anthracis* naturally infects mammals such as cows and sheep as well as humans, it is reasonable to use a mouse infection model of *B. anthracis*. Koch determined three principles, referred to as Koch's postulates, for identifying infectious disease‐causing pathogens. One of these three principles is that a pure cultured microorganism causes the disease in healthy susceptible animals. Since Koch's study, several mammals that are phylogenetically close to humans have been used as healthy susceptible animals. In the dawning age of infection research around the late 19th century, the aims of animal infection experiments were to identify the causative pathogens and toxins, and evaluate antisera and vaccines. In the present day, genome science has revealed a vast number of biologic molecules that constitute pathogenic microorganisms and their host animals, prompting investigations of their functions in infectious processes. Furthermore, the biomolecules found to be involved in the infectious processes are new targets for anti‐infective drugs. Therefore, current infection research requires a large number of experiments.

Infection experiments using mammals are costly. For example, an inexpensive mouse strain costs approximately 10 US dollars per mouse. In addition, the use of mammals for research purposes is restricted internationally from an ethical point of view.^1^ To perform infection experiments using mammals, research‐planning documents must adhere to international guidelines and be approved by the institutional ethics committee. The high cost and ethical issues regarding the use of mammals for research can mostly be avoided when non‐mammalian animals are used. Some review articles describe a specific non‐mammalian animal model for infection studies, but few review articles broadly describing non‐mammalian animal infection models are available to help researchers select the most appropriate non‐mammalian model for their specific experimental purposes. In this review, we summarize the advantages of non‐mammalian infection models and describe several non‐mammalian models recently used to study infection.

## 2 CRITERIA FOR ANIMAL INFECTION MODELS

Animals used for infection experiments should satisfy the following criteria. Researchers must select an animal species whose characteristics are most suitable for the experimental purpose.

### 2.1 Obtaining animals with homogeneous conditions

A large number of animals is needed for evaluating the virulence activity of a bacterial strain. Animals with different conditions, such as maturation, body weight, and health level, will exhibit different susceptibility to bacteria. Thus, ideally, researchers should obtain a large number of animals with homogeneous conditions. Animals can be raised in a laboratory or are commercially available. For insect models, however, researchers can easily obtain animals whose body weight is almost the same by selecting animals that underwent molting at about the same time.

### 2.2 Space for breeding and infection experiment

A large space for breeding and infection experiments requires a lot of effort to maintain. A small space is desirable to maintain animal health and to perform infection experiments. Autoclavable and disposable plastic containers are convenient for infection experiments.

#### 2.2.1 Injection

For infection experiments, a specific amount of bacterial solution is injected into a specific part of the animal body. If the animal is large enough to use a medical syringe and needle, it is easy to conduct quantitative injection experiments. In addition, animals that can be controlled without anesthesia or a holding apparatus are easier to use for infection experiments.

### 2.3 Research on host factors

To investigate the host factors involved in infectious processes, it is important to perform a biochemical approach by purifying biologic molecules, as well as a genetic approach by creating genetically modified animals. In the past, genetic modification was performed only in model organisms such as *Caenorhabditis elegans*, *Drosophila melanogaster*, and mice. Recently, genome editing technology has enabled genetic modifications in many animal species. Small‐sized animals are generally not suitable for purifying biomolecules due to the small amount of biologic starting material. But when the target biomolecules are expressed in high amounts, small‐sized animals could be used for a biochemical approach.

## 3 NON‐MAMMALS USED AS INFECTION MODELS

The characteristics of non‐mammals used as infection models of human pathogenic bacteria are described below.

### 3.1 *Caenorhabditis elegans*

*C. elegans* is an important model organism in embryology in which all cell lineages can be pursued. In the late 1990s, Ausubel *et al.* began utilizing this animal as an animal infection model of *Pseudomonas aeruginosa*.^2^ This is the first case in which a non‐mammal was used as an infection model of a human pathogen. *C. elegans* is normally fed *Escherichia coli*. When *C. elegans* is fed pathogenic bacteria such as *P. aeruginosa*, the *C. elegans* dies. There are two known killing mechanisms: toxin secreted from the bacteria kills the animal and digesting the bacteria kills the animal.^3^ When bacterial infection kills *C. elegans*, it is difficult to estimate the number of infected bacteria in the *C. elegans* because the infection inhibits feeding behavior. Genetic manipulation methods are well established in *C. elegans*, enabling genetic analysis of host factors. Due to its small body size, it is easy to maintain the worms in a small space, but it is difficult to inject a bacterial solution into the worm (Table 1). *C. elegans* can be obtained from genetic stock centers or other researchers, and can be proliferated in a laboratory. *C. elegans* has been used in infection experiments with various human pathogenic bacteria such as *S. aureus* and *Klebsiella pneumoniae* (Table 2).

**Table 1:** Characteristics of non‐mammalian infection models and mouse infection model

	Cost^a^	Space	Injection^b^	Research on host factor	
Model animal				Genetic mutant^c^	Biological material
*C. elegans*	Low	Small	Difficult	Available	Small
Silkworm (*B. mori*)	Low	Small	Easy	Non‐available	Large
Fruit fly (*D. melanogaster*)	Low	Small	Difficult	Available	Small
Zebrafish (*D. rerio*)	Middle	Small	Normal/Difficult^d^	Available	Large
Two‐spotted cricket (*G. bimaculatus*)	Low	Small	Easy	Non‐available	Large
Hornworm (*M. sexta*)	Low	Small	Normal	Non‐available	Large
Waxworm (*G. mellonella*)	Low	Small	Normal	Non‐available	Large
Mouse (*M. musculus*)	High	Large	Normal	Available	Large

a Low, less than 1 US dollar/animal; Middle, 1–5 US dollars/animal; High, more than 5 US dollars/animal.

b Easy, requires no anesthesia, holding apparatus, or microscope; Normal, requires anesthesia or holding apparatus, but no microscope; Difficult, requires glass capillary and microscope.

c Available means that genetically modified animals can be obtained from a genetic stock center.

d Adult fish, Normal; embryo, Difficult.

**Table 2:** Bacterial species evaluated in non‐mammalian infection models

Bacterial species	*C. elegans*	Silkworm	Fruit fly	Zebrafish	Two‐spotted cricket	Hornworm	Waxworm
*Campylobacter jejuni*	−	−	−	−	−	−	Yes^36^
*Francisella tularensis*	Yes^37^	Yes^38^	Yes^9^	Yes^39^	−	−	Yes^40^
*Legionella pneumophila*	Yes^41^	−	Yes^42^	−	−	−	Yes^43^
*Acinetobacter baumannii*	Yes^44^	−	−	Yes^45^	−	−	Yes^24^
*Pseudomonas aeruginosa*	Yes^2^	Yes^4^	Yes^46^	Yes^47^	Yes^12^	Yes^48^	Yes^49^
*Klebsiella pneumoniae*	Yes^50^	−	Yes^51^	Yes^52^	−	−	Yes^53^
*Shigella* sp.	Yes^54^	−	−	Yes^55^	−	−	Yes^56^
*Yersinia pestis*	Yes^57^	−	−	−	−	−	Yes^58^
*Mycobacterium* sp.	Yes^59^	Yes^60^	Yes^61^	Yes^11^	−	−	Yes^62^
*Enterococcus faecalis*	Yes^63^	−	Yes^64^	Yes^65^	−	Yes^66^	Yes^67^
*Staphylococcus aureus*	Yes^63^	Yes^4^	Yes^7^	Yes^68^	Yes^12^	Yes^15^	Yes^69^
*Streptococcus pyogenes*	Yes^63^	Yes^26^	−	Yes^10^	−	−	Yes^70^
*Listeria monocytogenes*	Yes^71^	Yes^72^	Yes^73^	Yes^74^	Yes^12^	−	Yes^75^
*Bacillus cereus*	Yes^76^	Yes^77^	Yes^78^	−	−	−	Yes^79^

− indicates no report available.

### 3.2 **Silkworm (** *Bombyx mori* **)**

**Figure 1:**
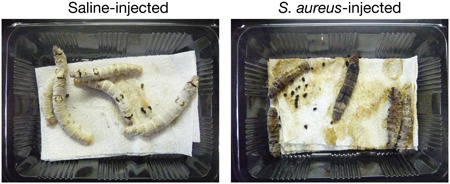
Silkworm *Staphylococcus aureus* infection model. Silkworms are injected into the hemolymph with *S. aureus* (10^7^ CFU/larva) or saline. Images obtained 3 days after infection are shown. All the larvae injected with *S. aureus* died. [Color figure can be viewed at wileyonlinelibrary.com]

The silkworm is the larva of *Bombyx mori*, a lepidopteran insect that has been utilized in the silk industry for more than 4000 years. In 2002, we found that various human pathogenic bacteria, including *S. aureus*, kill silkworms (Figure 1).^4^ Because the silkworm has a large body size and is slow‐moving, it is easy to inject an appropriate amount of sample solution into the silkworm hemolymph using a 27‐gauge needle and syringe without anesthesia^5^ (Table 1). This accurate injection technique enables the evaluation of bacterial virulence properties by determining the median lethal dose.^5^, ^6^ Skilled researchers can inject 150 silkworms in 1 hr. Silkworm eggs, larvae, and artificial diets are commercially available in various regions of the world, including Europe, the United States, and Japan. Because the silkworm is a domesticated animal that cannot proliferate in nature and does not escape from its cage, the silkworm infection model is suitable for experiments using biohazardous infectious agents. The silkworm has been used as an infection model of various human pathogenic bacteria such as *P. aeruginosa* and *S. pyogenes* (Table 2).

### 3.3 **Fruit fly (** *Drosophila melanogaster* **)**

The fruit fly is used as a model organism for genetic analysis. The Toll‐like receptor, an important receptor in the innate immune system, was discovered in a fruit fly infection model with *E. coli*. The fruit fly has been used as an infection model for many human pathogenic bacteria, including *S. aureus*.^7^ To perform infection experiments using the fruit fly, anesthesia is induced by carbon dioxide gas and a needle dipped in bacterial solution is used to cause injury under a microscope, or the fly can be injected with bacterial solution using a glass capillary under a microscope (Table 1).^8^, ^9^ Biohazard prevention requires considerable attention, because flies can escape by flying away. Fruit flies can be obtained from stock centers or other researchers, and can be proliferated in a laboratory.

### 3.4 **Zebrafish (** *Danio rerio* **)**

The zebrafish is a well‐known aquarium fish and model organism. The embryo is transparent and thus suitable for observing organ development. Both adult fish and embryos are used for infection experiments (Table 1). To infect an adult fish, the fish is anesthetized with tricaine and injected with a bacterial solution using a syringe and a 29‐gauge needle.^10^ To infect an embryo, the bacterial solution is injected using a glass capillary under a microscope.^11^ The transparency of the embryo enables real‐time imaging of the infectious process by *Mycobacterium* ^11^ (Table 2). Zebrafish can be purchased from ornamental fish shops, or the fish strain used for genetic analysis can be obtained from genetic stock centers or other researchers. It should be noted that fish experiments now require approval from research ethics committees.

### 3.5 Two‐spotted crickets

**Figure 2:**
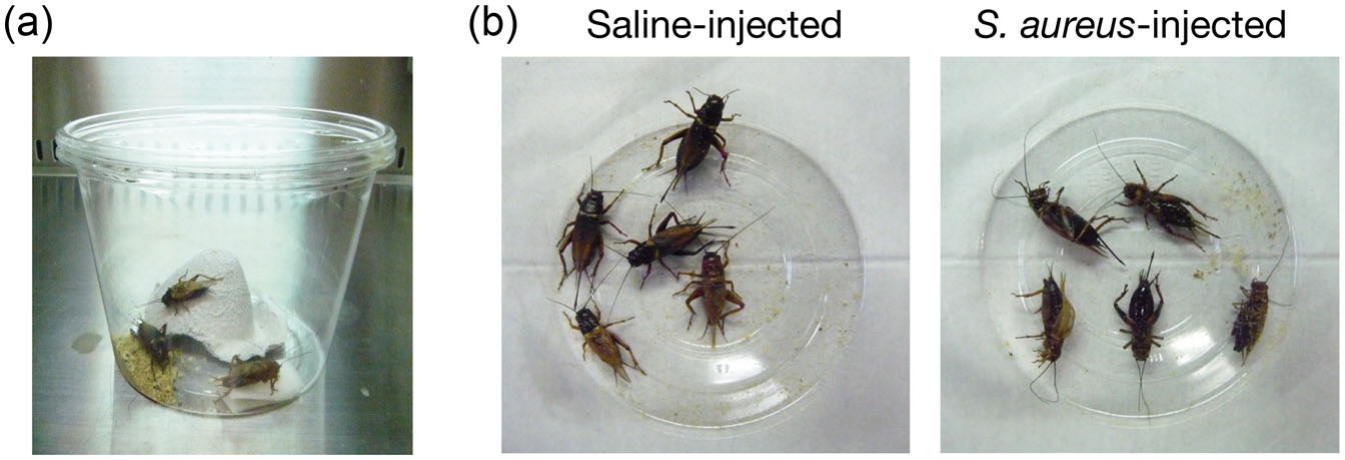
Infection model using the two‐spotted cricket. (a) A disposable plastic cage (diameter 130 mm × height 100 mm) used for infection experiments with two‐spotted crickets is shown, in which five crickets are grouped together and provided cricket food, water, and shelter. (b) Two‐spotted crickets were injected with *Staphylococcus aureus* (10^8^ CFU/cricket) or saline. Images obtained 1 day after infection are shown. All the crickets injected with *S. aureus* died. [Color figure can be viewed at wileyonlinelibrary.com]

Crickets have long been used as model animals of calling behavior and aggressive behavior. Two‐spotted crickets and house crickets are easy to purchase and maintain, because these crickets are captive‐bred as food for reptiles. The two‐spotted cricket is a tropical insect that can be kept at 37°C, human body temperature. We examined two‐spotted crickets as an animal infection model of human pathogens and found that human pathogenic bacteria and fungi kill two‐spotted crickets^12^, ^13^ (Figure 2). Furthermore, we revealed that the sensitivity of two‐spotted crickets to *S. aureus* and *P. aeruginosa* is not different between 27°C and 37°C, whereas the sensitivity of two‐spotted crickets to *Listeria monocytogenes* is greater at 37°C than at 27°C.^12^ These findings suggest that two‐spotted crickets do not have increased sensitivity to all pathogens at a high temperature, but rather that specific pathogens exhibit increased virulence properties in high temperature conditions. Utilization of the two‐spotted cricket model allows for comparisons of the virulence of pathogenic bacteria at low and high temperatures, and identification of temperature‐dependent virulence mechanisms of bacteria. In infection experiments, two‐spotted crickets are injected with a bacterial solution using a syringe with a 30‐gauge needle without anesthesia^12^ (Table 1).

### 3.6 **Hornworm (** *Manduca sexta* **)**

The hornworm is a larva of *M. sexta*, a lepidopteran insect that damages tobacco leaves, and has been used for research on hormones and neurons. Hornworms are captive‐bred as food for reptiles or fish, and fertilized eggs and artificial foods are commercially available (Table 1). The hornworm was first examined as an animal infection model of *Bacillus cereus*, a human pathogenic bacterium^14^ and subsequently used for evaluating the virulence factors of other pathogens, such as *S. aureus* ^15^ and *Streptococcus pneumoniae* ^16^ (Table 2). After anesthetization by ice, hornworms are injected with bacterial solution using a Hamilton syringe with a 30‐gauge needle.^17^

### 3.7 **Waxworm (** *Galleria mellonella* **)**

The waxworm is a larva of *G. mellonella*, which feeds on honeybee nests. Waxworms are captive‐bred worldwide as food for amphibians, reptiles, and fish, and are easy to purchase and maintain (Table 1). For injection of waxworms, the worms are anesthetized on ice and injected using a syringe with a 30‐gauge needle,^18^, ^19^ or held in a special apparatus and injected.^20^ Because waxworms can grow at 37°C,^21^ infection experiments can be performed at 37°C. All human pathogens that have been examined in the waxworm infection model at 37°C, including *S. aureus*,^22^ *Cryptococcus neoformans*,^23^ and *Acinetobacter baumannii*,^24^ exhibited increased killing activity against waxworms at 37°C compared with a low temperature, raising the possibility that the waxworm immune system is damaged at 37°C and evaluation of the temperature effects on the virulence properties of pathogens should be evaluated cautiously in this model. A number of human pathogenic bacteria have been examined in the waxworm infection model (Table 2).

## 4 COVERAGE OF NON‐MAMMALIAN INFECTION MODEL IN INFECTION RESEARCH

In infection experiments, animals must exhibit sensitivity against the pathogen. Whether a non‐mammal is susceptible to a human‐pathogenic bacterium depends on the conservation of the organ, tissue, cell, and cell signaling involved in the infection process between non‐mammals and mammals. The innate immune system constitutes the first defense mechanism against invading pathogens and is similar in many aspects between mammals and insects. For example, antimicrobial peptides that act as humoral antimicrobials, Toll‐like receptors that recognize pathogens by pattern recognition, and intracellular signaling pathways that are triggered by Toll‐like receptors are conserved between mammals and insects.^25^ In addition, although insect appearance is distinct from that of mammals, insects have compartments that function as the blood, gut, liver, kidney, and heart. Therefore, interactions between the pathogen and host that depend on conserved biologic systems can be analyzed in insect infection models. Various human pathogenic bacteria are analyzed in many insect infection models (Table 2). We previously reported that *S. aureus*, *P. aeruginosa*, *S. pyogenes*, and enterohemorrhagic *E. coli* kill silkworms^4^, ^26^ (Table 2), identified the bacterial genes required for killing silkworms, and revealed that the identified genes contribute to virulence against mammals.^6^, ^26^, ^27^, ^28^, ^29^, ^30^ Some of the bacterial genetic mutants with attenuated killing activity against silkworms are sensitive to oxidative stress in macrophages, antimicrobial peptides, and complements.^30^, ^31^ Because bacterial resistance to the host innate immune system is essential for bacterial infection in both mammals and insects, the silkworm infection model can be used to evaluate the virulence properties of human pathogenic bacteria. In contrast, there are differences between mammals and silkworms in the host–pathogen interaction. For example, neurotoxins for mammals, including botulinum toxin and morphine, are less toxic to silkworms, although both alpha and beta hemolysin of *S. aureus* are toxic to silkworms.^32^, ^33^ This difference could be due to the presence or absence of toxin receptors in silkworms. When pathogens infect animals *via* mammalian‐specific routes, non‐mammalian infection models cannot be used to evaluate bacterial virulence. Recent progress in genome editing technologies may solve such problems of non‐mammalian infection models by constructing humanized non‐mammalian models in future.

Chronic infection is a unique aspect of some human pathogenic bacteria such as *Mycobacterium tuberculosis*. At present, most non‐mammalian infection models are used for acute infection by human pathogenic bacteria, in which bacteria proliferate in hemolymph or blood and kill animals within 10 days. Chronic infection experiments using non‐mammalian models are not well established and challenging. In the zebrafish model, chronic infection by *Mycobacterium marinum* has been studied as a model of *M. tuberculosis* infection in humans. *M. marinum* infection in adult zebrafish is prolonged for more than 8 weeks and forms granulomas, a characteristic of chronic tuberculosis infection.^34^, ^35^

## 5 CONCLUDING REMARKS

This review outlines non‐mammalian infection models used over the past two decades. In the absence of an appropriate established infection model among current infection models, a new animal infection model must be established to proceed with the infection research. Most of the non‐mammals mentioned in this review are utilized as foods for animals such as reptiles and fish. Food animals are easily maintained and bred, grow rapidly, have large bodies, and are inexpensive, which are desirable characteristics for animal infection models. Various non‐mammalian species that are used as food animals are good resources for establishing new animal infection models and will be powerful tools for infection research.

## CONFLICT OF INTEREST

All authors declare there are no conflicts of interest.

## Data Availability

Data sharing not applicable to this article as no datasets were generated or analyzed during the current study.
